# Human Cough as a Two-Stage Jet and Its Role in Particle Transport

**DOI:** 10.1371/journal.pone.0169235

**Published:** 2017-01-03

**Authors:** Jianjian Wei, Yuguo Li

**Affiliations:** 1 Department of Mechanical Engineering, The University of Hong Kong, Hong Kong; 2 Shenzhen Institute of Research and Innovation, Shenzhen, China; Coastal Carolina University, UNITED STATES

## Abstract

The human cough is a significant vector in the transmission of respiratory diseases in indoor environments. The cough flow is characterized as a two-stage jet; specifically, the starting jet (when the cough starts and flow is released) and interrupted jet (after the source supply is terminated). During the starting-jet stage, the flow rate is a function of time; three temporal profiles of the exit velocity (pulsation, sinusoidal and real-cough) were investigated in this study, and our results showed that the cough flow’s maximum penetration distance was in the range of a 50.6–85.5 opening diameter (*D*) under our experimental conditions. The real-cough and sinusoidal cases exhibited greater penetration ability than the pulsation cases under the same characteristic Reynolds number (Re_c_) and normalized cough expired volume (*Q/AD*, with *Q* as the cough expired volume and *A* as the opening area). However, the effects of Re_c_ and *Q*/*AD* on the maximum penetration distances proved to be more significant; larger values of Re_c_ and *Q*/*AD* reflected cough flows with greater penetration distances. A protocol was developed to scale the particle experiments between the prototype in air, and the model in water. The water tank experiments revealed that although medium and large particles deposit readily, their maximum spread distance is similar to that of small particles. Moreover, the leading vortex plays an important role in enhancing particle transport.

## Introduction

The human cough is known to be a significant vector for transmitting respiratory diseases in indoor environments. Thousands of droplets per respiration can be released during breathing, coughing and sneezing. Once exhaled, droplets evaporate and become droplet nuclei [1]. These droplets and droplet nuclei can contain elements such as sodium, potassium and chloride in solutes; DNA, lipids, glycoproteins and proteins in suspended insoluble solids; and, of course, infectious pathogens if released by an infectious patient. Exposure to these pathogen-containing droplets can occur via both short- (within 1–2 m of the source patient) and long- (beyond about 2 m in the indoor environment) range routes. The former is known as direct spray infection [2], in which relatively large (≥ 5 μm in diameter) droplets or droplet nuclei can be directly deposited on the nasal or oral mucosa of the new host. Short-range airborne exposure via smaller droplets or droplet nuclei is also important in close proximity infection [3]. Beyond 1–2 m, the exhaled air stream dissolves into the room airflow, and the pathogen-containing droplets or droplet nuclei are dispersed according to the global airflow in the room. In general, much less is known about the mechanism and control of short-range airborne exposure than about long-range airborne routes.

Coughing has been more extensively investigated than breathing and sneezing in disease transmission [4]. The number of droplets during a single cough can be as high as 3,000 [4], with varying totals among different experiments [5–8]. Wells [1] first defined large droplets as those over 100 μm in aerodynamic diameter. There is one peak of the droplet number concentration in the sub-micron range and another peak at over 10 μm [7, 9]. The travel distance of the cough airflow, taken with the dispersion characteristics of expired droplets, are of particular interest [10]. Such information is essential for taking appropriate action to reduce or eliminate the probability of infection in both community and health care environments.

In terms of experimental studies, the transient velocity distribution and width of cough airflow have been measured using methods such as the particle image velocimetry (PIV) technique. A maximum velocity range of 6–28 m/s has been detected, and the cough was found to expand linearly in the initial stage [6, 7, 11–13]. Tang [14] estimated the maximum velocity to be 8 m/s using the Schlieren imaging technique. Gupta et al. [15] showed that the cough flow rate can be represented as a combination of gamma-probability-distribution functions. Zhu et al. [6] visualized the coughing dispersion process and found that the cough airflow might travel farther than 2 m.

In terms of computational and modeling studies, a number of CFD simulations [16–19] and experimental observations on manikins [20, 21] have been conducted, and cough-related expiratory droplet dispersion in complex indoor environments has also been investigated. To simplify the complex coughing phenomenon, the cough is sometimes approximated as a steady jet [10]. However, Rim and Novoselac [22] compared short-term release of particles in the transient jet and continuous release of particles in a steady jet and found that higher exposure was caused by the latter. Villafruela et al. [21] compared the transient and steady boundary conditions for breathing, and learned that the transient jet with a sinusoidal function does not penetrate as far as the steady jet under the mixing ventilation strategy. In addition, the transient cough is characterized by a leading vortex and its trailing flow [14, 23]; thus, the vortex structure might be important in particle transport but absent in the steady jet [24]. A cough of infinte duration is close to a steady jet; thus, the duration of the cough is important in determing its penetration property and the associated particle transport.

**Fig 1:**
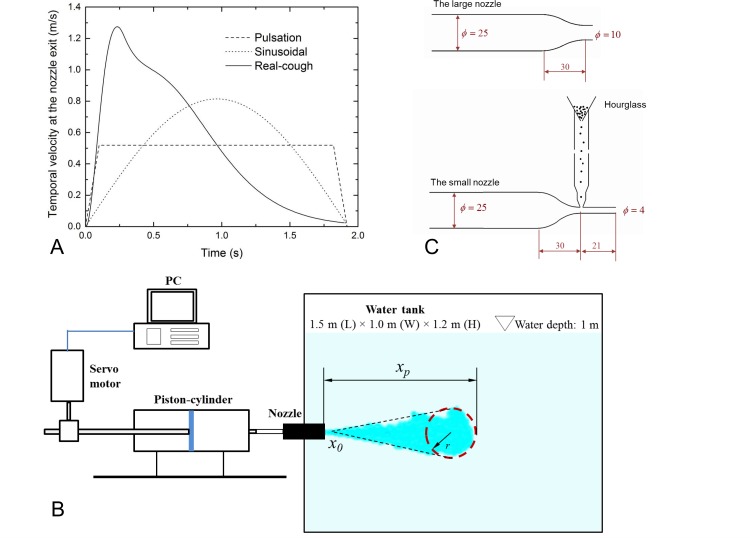
Experimental set-up. (A) Three temporal profiles at the nozzle exit investigated in this study; (B) schematic diagram of the test apparatus; and (C) the two nozzles used in this study (unit: mm). The large nozzle (*D* = 10 mm) was for the food dye experiments, and the size of the small nozzle (*D* = 4 mm) was chosen by using the similarity protocol for the particle experiments. *x*_*p*_ is the streamwise penetration distance, and *x*_*0*_ is the virtual origin.

In contrast, it has also been shown that a cough cannot be characterized by a simple puff, which is a sudden release of finite fluid with a normalized expired volume below 100 [11, 25–27]. One cough produces between 0.6 and 1.6 L of airflow at a peak velocity of about 10 m/s, with a typical duration of 0.5 s; thus, the normalized expired volume lies in the range of 100 to 250 if the mouth diameter is 0.02 m [11, 15]. The cough flow presents the starting-jet property, after which it becomes an interrupted jet. Classic fluid mechanics theories on starting and interrupted jets [22, 28–30] provide insights into cough flow development. Hypothetically, these theories can be easily extended to cough flows [31]. However, the existing studies have only dealt with simple temporal exit velocity, such as the pulsation profile, whereas a real cough exhibits a more complex temporal velocity profile approximated as a combination of gamma-probability-distribution functions [15] (see Fig 1A). More studies on the effects of cough boundary conditions and the dynamic development of the cough flow are necessary.

Our objective was to perform an experimental study of the two-stage cough jet and investigate the effects that different boundary conditions such as temporal exit velocity profiles, cough duration and velocity scale have on cough flow penetration. The exhaled particles spread by the cough flow were also investigated.

## Methods

The experiments were conducted in a rectangular water tank (see Fig 1B) 1.5 m (length) × 1 m (width) × 1.2 m (height) in dimension. A supply nozzle discharged dyed or particle-filled water into the tank horizontally at a height of 0.5 m. Two converging nozzles were used, as shown in Fig 1C. A large nozzle (*D* = 10 mm) was adapted from Longmire and Eaton [32] for the food dye (royal blue, by Americolor) experiments to ensure a uniform velocity distribution at the nozzle exit. A small nozzle (*D* = 4 mm), equipped with a sediment feeding system as adapted from Li [33], was used to test the particles transported by the discharged fluid. Glass beads (ρ = 2480 kg/m^3^) of three size categories were used: small (30–50 μm, Polysciences Inc., category no 18901–100), medium (210–250 μm, 18902–100) and large (355–420 μm, 18905–100) particles. The particle volume ratio in the modelled cough flow was below 0.5% to ensure that the flow was not affected by the adding of particles. The protocol for scaling particle experiments between air and water are described later. The source fluid was supplied to the nozzles using a piston-cylinder driven by the programmable servo motor system (Kollmorgen AKM 24F, AKD-P00606 and NI PCI-7342, UMI-7772).

The test conditions of the food dye experiments are summarized in Table 1. Three temporal exit velocity profiles were investigated, including two simplified profiles (i.e., pulsating and sinusoidal) and a real-cough profile. The real-human cough flow rate is represented as a combination of gamma-distribution-probability functions, as shown by Gupta et al. [15].

**Table 1:** Summary of data on cough flow penetration distances. *t*_*inject*_ is the injection duration in the starting jet stage, *t*_max_ is the time when *dx*/*dt* < 0.01 m/s and the cough flow is considered to reach the maximum distance.

CaseNo.	Boundary conditions			Streamwise penetration distances			
	Temporal velocity profiles	Rec=UcDν	*Q*/*AD*	$\frac{x_{p1}}{D}$	$\frac{x_{p2}}{D}$	$\frac{t_{\max}}{t_{inject}}$	$\frac{x_{p,\max}}{D}$
1	Pulsation	5,200	100	23.5	39.5	5.7	50.6
2		7,900	150	29.6	50.9	6.7	71.0
3		12,900	150	27.4	55.2	13.2	80.9
4		12,900	250	37.5	62.9	7.7	85.5
5	Sinusoidal	5,200	100	27.6	44.7	5.8	55.7
6		5,200	150	35.2	57.3	3.8	68
7		7,900	150	34.8	57	6.7	79.3
8	Real-cough	5,200	100	30.7	43.7	5.7	53.4
9		5,200	150	40.2	58.8	3.8	67.5
10		7,900	150	38.2	54.9	6.5	69.7

Note: *x*_*p1*_ and *x*_*p2*_ are the respective streamwise penetration distances at *t* = *t*_max_ and *t* = 3*t*_max_, respectively. The cough flow reaches its maximum penetration distance *x*_p,max_ at *t*_max_ if its penetration velocity drops below 0.01 m/s.

The average injection velocity is used as the characteristic velocity.

$$U_{c}=\frac{1}{t_{inj}}\int_{0}^{t_{inj}}U(t)dt$$ (1)

where *t*_*inj*_ is the cough duration, or the duration of the starting-jet stage.

For the sinusoidal profile, the peak velocity during the starting-jet stage is *U*_max_ = 1.57*U*_*c*_, and for the real-cough profile is *U*_max_ = 2.49*U*_*c*_.

The characteristic Reynolds number is defined as

Rec=UcDν (2)

where *ν* is the kinematic viscosity of the fluid. The Reynolds number is in the range of 5,200 to 12,900, with normalized expired volumes varying between 100 and 250. The water temperature for the food dye experiment was maintained close to 20°C (*ν* = 1.004×10−^6^ m^2^/s).

The motion of the dyed liquid was recorded as a function of time using a Canon 6D camera with a 24–105 mm focal lens at 50 frames per second. The video records were analyzed to provide the maximum streamwise penetration distances (*x*_*p*_) of the cough flow. Each case was repeated three times and averaged. The standard deviation in streamwise distances was less than 6%.

In the particle experiment, the mid-sagittal plane of the nozzle was illuminated by a 3-mm laser sheet produced by a 3W DPSS 532 nm laser projector (Ourslux Lighting Technology Co, Ltd). The Canon 6D camera described above was used, and a series of pictures were blended with Startrails.exe to obtain the streak image of particles.

The protocol for scaling particle experiments between air and water is as follows. This is the first implementation of such a protocol, to the best of the authors’ knowledge. The density ratio of particles to fluid is much smaller in water than in air, which causes some difficulty when quantitatively mapping water tank experimental data to the real scenario in air. However, agreement between the two is possible if we make some assumptions, e.g., ignoring the non-linearity of the drag force and ignoring the force due to fluid acceleration, added mass force and Basset history force; all of which are plausible if the particle density is much larger than that of water [34].

Under the Stokes’ region (Rep=|uf−up|dpν<1), the law of particle motion is

$$\frac{du_{p}}{dt}=\frac{1}{\tau }(u_{f}-u_{p}-u_{\tau })$$ (3)

where ***u***_*τ*_ is the terminal settling velocity of particles and *τ* is the particle relaxation time. They are given by

$$u_{\tau }=\frac{\left(\rho_{p}-\rho_{f}\right)d_{p}^{2}g}{18\mu },\;\tau =\frac{\rho_{p}d_{p}^{2}}{18\mu }$$ (4)

Normalizing Eq (3) by the characteristic length scale *D* and velocity scale *U*_*c*_ gives us

$$\frac{d{\bar{u}}_{p}}{d\bar{t}}=\frac{1}{St}\left({\bar{u}}_{f}-{\bar{u}}_{p}-{\bar{u}}_{\tau }\right)$$ (5)

where $St_{c}=\frac{U_{c}\tau }{D}$ is the Stokes number.

In water tank modeling, the same *Re* is essential to ensure the dynamic similarities between the velocity fields of air and water (${\bar{u}}_{f}$),

Rec=Uc,gDgνg=Uc,wDwνw (6)

where the subscript *g* stands for air, and *w* for water.

According to Eq (5), we need to satisfy two more conditions to make particle motions comparable in the two systems

$$St_{c,g}=St_{c,w}$$ (7)

$$\frac{u_{\tau ,g}}{U_{c,g}}=\frac{u_{\tau ,w}}{U_{c,w}}$$ (8)

Combining Eqs (4), (7) and (8) gives us

$$\frac{U_{c,g}^{2}}{D_{g}}\frac{\rho_{p,g}}{\rho_{p,g}-\rho_{g}}=\frac{U_{c,w}^{2}}{D_{w}}\frac{\rho_{p,w}}{\rho_{p,w}-\rho_{w}}$$ (9)

Combining the scaling laws described by Eqs (6) and (9) gives us

$$\frac{U_{c,w}}{U_{c,g}}=\sqrt[{3}]{\frac{\nu_{w}}{\nu_{g}}\frac{\rho_{p,g}}{\rho_{p,g}-\rho_{g}}\frac{\rho_{p,w}-\rho_{w}}{\rho_{p,w}}}$$ (10)

$$\frac{D_{w}}{D_{g}}=\sqrt[{3}]{{\left(\frac{\nu_{w}}{\nu_{g}}\right)}^{2}{\left(\frac{\rho_{p,g}}{\rho_{p,g}-\rho_{g}}\frac{\rho_{p,w}-\rho_{w}}{\rho_{p,w}}\right)}^{-1}}$$ (11)

Eqs (10) and (11) define the geometric and boundary conditions for particle experiments in water tanks (e.g., the nozzle diameter and exit velocity). The particle size is regulated by Eqs (4) and (8).

The water temperature was maintained close to 20°C, so *U*_*c*,*w*_/*U*_*c*,*g*_ = 0.35, *D*_*w*_/*D*_*g*_ = 0.20 according to Eqs (10) and (11). The nozzle diameter (4 mm) corresponds to a mouth diameter of 2 cm for human coughing. As the injection period with the small nozzle is quite short (about 0.3 s), it became impossible to implement the real-cough or sinusoidal profile with the servo motor. Only the pulsation profile was investigated with the small nozzle.

Glass beads of three size categories were used in this experiment, as described above. The analogous particle sizes in realistic human coughing are as follows: 8–14 μm (small particles), 57–68 μm (medium particles) and 96–114 μm (large particles), according to Eqs (4) and (8).

## Results

### Visualization of the two-stage cough flow

**Fig 2:**
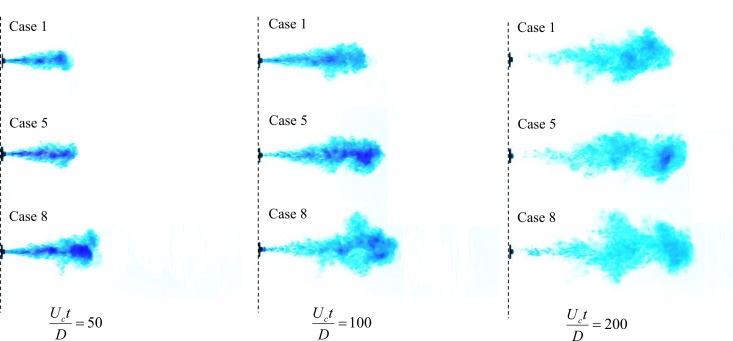
Visualizations of the turbulent round starting and interrupted jets. Case 1 [pulsation], Case 5 [sinusoidal] and Case 8 [real-cough]. Re_c_ = 5200 and *Q*/*AD* = 100 for all cases. The flow transition from the starting to the interrupted jet stage occurred at *U*_*c*_*t*/*D* = 100 when the source supply is terminated.

The two-stage cough flow (e.g., the starting- and interrupted-jet stages) was visualized by a royal blue food dye, and photos were recorded and analyzed as a function of time, as discussed in the Methods section. The visualizations of Cases 1 [pulsation profile], 5 [sinusoidal profile] and 8 [real-cough profile] are shown in Fig 2. Re = 5200 and *Q*/*AD* = 100 for these three cases, which were equal in total injected mass of fluid and injection times. It is clear from Fig 2 that the flow with a real-cough profile penetrated the ambient fluid at the greatest velocity in the starting-jet stage; however, it gradually lost its advantage in the interrupted-jet stage compared with the sinusoidal case. The probable reason is the trailing flow cannot efficiently supply momentum to the leading vortex in the real-cough case. The tail in the real-cough case was too slow to catch up with the main flow. As a result, the interrupted jet had a longer length than in the pulsation case (Case 1).

### Development of the two-stage cough flow

**Fig 3:**
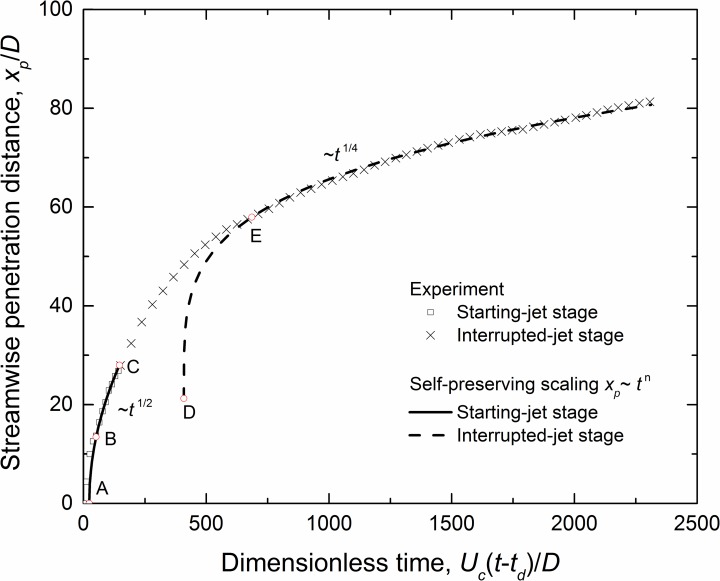
Streamwise penetration distance as a function of time in the complete process of Case 3 [Pulsation, Re = 12900, *Q*/*AD* = 150].

The cough’s expired flow entrains ambient fluid as it travels, increasing its size and decreasing its speed. It presents a self-preserving manner, as described by Morton et al. [35]. The cough jet’s complete propagation process in Case 3 [Pulsation, Re = 12900, *Q*/*AD* = 150] is shown in Fig 3. The injection is started at *t* = 0 and interrupted at Point C. The starting jet takes on the self-preserving property at point B, with the virtual origin indicated by point A. After the injection is interrupted, the flow keeps traveling, but the transition from *t*^1/2^ to *t*^1/4^ takes a period (C to E). The virtual origin of the interrupted jet is indicated by Point D.

**Fig 4:**
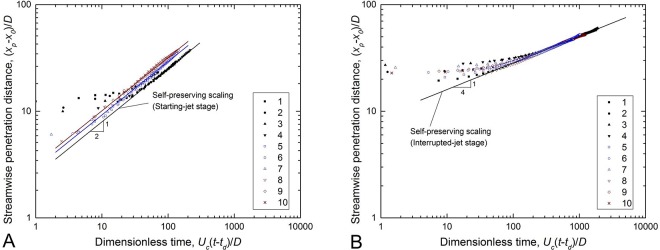
Streamwise penetration distances of the jet tips as a function of time. (A) starting-jet stage; (B) interrupted-jet stage.

The normalized streamwise penetration distances of the cough flow are plotted in Fig 4A. The measured data follow the self-preserving correlation reasonably (R^2^ > 0.99), as described in Eq (12),

$$\frac{x-x_{0}}{D}=C_{x1}{\left[\frac{U_{c}\left(t-t_{d}\right)}{D}\right]}^{1/2}$$ (12a)

$$\frac{x-x_{0}}{D}=C_{x2}{\left[\frac{U_{c}(t-t_{d})}{D}\right]}^{1/4}$$ (12b)

where, *x* is the streamwise penetration distance of the jet tip; *t* is the time, and *t* = 0 s is when the jet is started; *x*_0_ is the virtual origin of the jet; *x*_*0*_/*D* is 0–2.7 in the starting-jet stage and 6.6–28.6 in the interrupted-jet stage; *t*_*d*_ is the extrapolated temporal origin of flow initiation; and *U*_*c*_*t*_*d*_/*D* is 9.4–23.3 and 72–468 for the two stages, respectively. *C*_*x1*_ and *C*_*x2*_ are the respective coefficients in the starting- and interrupted-jet stages, indicating the flow’s penetration ability. The experimentally obtained values of *C*_*x1*_ are 2.5, 2.9 and 3.2, respectively, for the pulsation, sinusoidal and real-cough profiles. *C*_*x2*_ = 9.0 for the interrupted-jet stage.

During the starting-jet stage, the total momentum supply is as follows

$$I={\int_{0}^{t_{inject}}\rho \frac{\pi }{4}D^{2}\left[U(t)\right]}^{2}dt$$ (13)

Cases 5 and 8 have the same Re_*c*_ and *Q*/*AD* as Case 1, but their momentum supplies are 23% and 62% higher, respectively. As a result, cough flow with a real-cough temporal profile (e.g., Case 8) has the strongest penetration ability, as it has the maximum momentum supply. The cough flow with a pulsation profile travels at the slowest speed (*C*_*x1*_ = 2.5 for the pulsation profile), which is close to the values in previous studies (e.g., 2.6 [28] and 2.5–3.2 [36]).

The cough jet can be characterized by a leading vortex and its trailing flow [23]. After the source fluid is terminated, the leading vortex and the trailing fluid continue to penetrate the still ambient fluid. However, as there is no more momentum supply, the velocity decay in this phase is greater than that in the starting jet stage, which is evident from the scaling law described by Eq (12). Despite the differences in temporal exit velocity profiles, all of the cases collapsed into the same self-preserving fitting, as shown in Fig 4B. The value of *C*_*x2*_ obtained in this study is close to the value of 8.0 obtained by Sangras et al. [28].

Once the penetration velocity drops below 0.01 m/s, the flow is subject to the ambient fluid field (e.g., ventilation and the human body thermal plume, etc.). This is referred to as the cough flow’s maximum penetration distance, which is summarized in Table 1. The maximum penetration distances in all of the tested cases were in the 50.6–85.5 *D* range. The temporal exit velocity profile had some effect on the maximum penetration distances. For example, the maximum penetration distances of the cough flow in Cases 5 [sinusoidal] and 8 [real-cough] were 10.1% and 5.5%, respectively—higher than that in Case 1 [pulsation]. However, the effect of the temporal profile was not as significant as those of *Q*/*AD* and Re_c_. Take Cases 5–7 for example. Increasing *Q*/*AD* alone from 100 to 150 (as in Cases 5 and 6) increased the maximum penetration distance by 22.1%. Increasing the Re_c_ from 5200 to 7900 (as in Cases 6 and 7) also raised the maximum penetration distance by 16.6%. A larger increase in traveling distances is expected with a further increase in *Q*/*AD* or Re_c_.

### Particle transport

In the particle transport experiment, the same Re_c_ and *Q*/*AD* as in Case 4 [Pulsation, Re = 12900, *Q*/*AD* = 250] were adopted. The injection duration was just 0.30 s, so only the pulsation profile was investigated. A long starting jet lasting 6 s is also considered in the Discussion section to compare the effects of injection duration.

**Fig 5:**
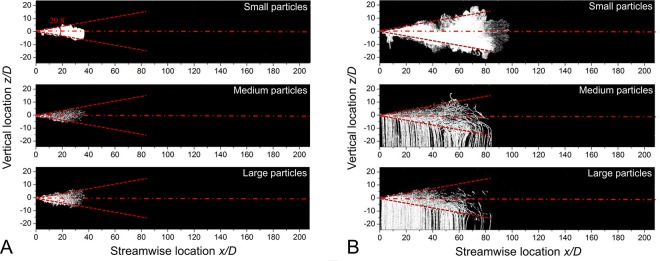
Streak pictures of particles in Case 4 [Pulsation, Re = 12900, *Q*/*AD* = 250]. The jet boundary is indicated by the red dashed line. The pictures overlap from *t* = 0 to (A) the time when the jet is interrupted (*t* = *t*_*inj*_), and (B) *t* = 10*t*_*inj*_.

Particles in three size categories were seeded into the discharged fluid about 5 *D* before the nozzle outlet (Fig 1C). Their streak pictures are shown in dimensionless form (normalized by *D*) in Fig 5. At the moment the source fluid interrupted, there is no obvious difference in their dispersion pattern (Fig 5A). The large particles seem to fill in the whole jet region, and few of them settle out the jet within such a short time. The maximum streamwise penetration distance of particles is about 38 *D*, the same as the jet tip listed in Table 1 at *t* = *t*_*inj*_. The cone-like particle-laden flow expands linearly with distance from the source, similar to the droplet cloud described by Bourouiba et al. [37].

**Fig 6:**
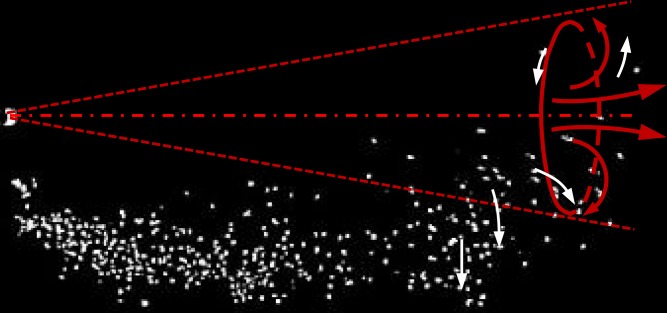
The instant dispersion pattern of large particles in the interrupted jet (*t* = 4.5*t*_*inj*_). The leading vortex is illustrated by red arrows, and white arrows indicate the particle motion.

At *t* = 10*t*_*inj*_, the particle cloud travels to a maximum distance of about 80 *D* (Fig 5B). These data can be scaled to the scenario in air using the protocol developed in this paper. In previous studies, the mouth opening area was given as 4 ± 0.95 cm^2^ for males and 3.37 ± 1.4 cm^2^ for females [15]. For the typical scenario in our study, the cough duration was 0.5 s in air (*U* = 10 m/s, *D* = 2 cm), such that small particles travelled about 1.6 m in 4.5 s after the cough stopped. Compared with the distance of 0.76 m in the first 0.5 s, the travel velocity was rather slow. Small droplets remained within the jet while medium and large droplets escaped due to gravitational force and continued to be deposited. Unlike previous studies (e.g., Xie et al., [10]), the maximum travel distance of medium particles in our study was similar to that of small particles. One probable reason lies in the rapid velocity decay of the interrupted jet. Although the small particles remained in the jet, they resolved into the ambient flow before travelling very far. The second reason is the leading vortex, the existence of which in the cough jet has been previously demonstrated by researchers (e.g., Tang et al. [38] and Bourouiba et al. [37]). The leading vortex, especially the upper vortex that provides a positive vertical velocity component, can trap the particles and carry them forward. Particles fall out of the jet after they are thrown into the trailing flow (see Fig 6).

## Discussion

### Influence of boundary conditions on the cough flow’s penetration distance

The boundary conditions (e.g., temporal exit velocity profile, Re_c_ and *Q*/*AD*) are important in determining the spread of the cough flow and pathogen-laden particles. The cough is an interrupted jet after the source supply is terminated. Bourouiba et al. [37] and Sangras et al. [28] emphasized the puff or interrupted-jet stage, but this study shows that the starting-jet stage is also quite important, to the extent that its consideration is necessary to understand cough dynamics and associated pathogen spread.

In realistic human coughs, *Q*/*AD* and Re correspond to the cough-expired volume of air and the velocity of the cough flow, respectively. Thus, the choice of these parameters is important in modelling cough flow dynamics. Specifically, there are two examples illustrating the effects of *Q*/*AD*.

**Fig 7:**
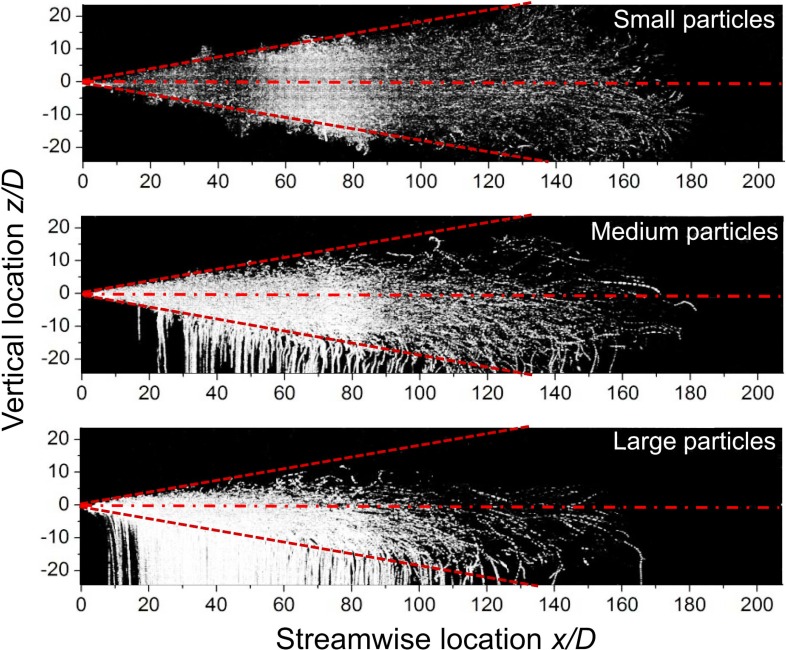
Particle streak lines from a long starting jet (Re_c_ = 12,900, *Q*/*AD* = 5,000).

First, if the injection duration is 20 times longer than in Case 4 (see Fig 5), the spread distance of particles is much enhanced, especially for small and medium particles, as shown in Fig 7. The difference in the fates of small and large particles is striking, with most of the large particles being deposited before reaching a distance of 120 *D*.

Pictures overlap from *t* = 0 until the jet is interrupted.

Second, the time needed for a fluid element to reach position *x* along the jet centerline is given by [39]

$$\frac{x(t)}{D}=\{\begin{array}{l} \;\frac{Ut}{D},\;x\leq 6.2D\\ 3.52{\left(\frac{Ut}{D}\text{-}3.1\right)}^{1/2},\;x>6.2D \end{array}$$ (14)

The coefficient is 3.52 for the steady jet, much larger than the value of 2.5 in our study for the pulsation cases. It is reasonable that the fluid element in the steady jet travels faster than in the starting jet, because the entrainment in the latter is stronger. The interrupted jet flow in Case 4 [Pulsation, Re = 12900, *Q*/*AD* = 250] only travelled to 85.5 *D*, whereas the steady jet with the same *U*_*c*_ travelled as far as 800 *D* before the velocity dropped below 0.01 m/s, according to Eq (14). Approximating a cough as a steady jet can introduce significant errors, as shown here.

### Implications for disease transmission in buildings

The most striking phenomenon observed in Fig 5 is that the particle clouds of all three sizes of particles penetrated almost the same distance at different time steps. The corresponding sizes in realistic coughing are 8–14 μm for small particles, 57–68 μm for medium particles and 96–114 μm for large particles. This is different from the steady jet assumption [10] where large particles were found to settle out of the cough jet early. We offer a two-part hypothesis to explain how large particles can reach a similar distance to that of fine particles. First, the cough duration is quite short, and the velocity of fine particles decays significantly after the jet is interrupted. Second, the leading vortex traps the particles; that is, as the leading vortex circulates and develops, particles are also trapped in the vortex.

It is unfortunate that the particle spread experiment could not be performed for the real-cough Cases 8–10, because the injection time was quite short. Based on the agreement of the particle penetration distance in Fig 5 and the cough penetration data for Case 4 in Table 1, we may reasonably assume that the particle penetration distance for the real-cough case would also agree with the data shown in Table 1 for Cases 8–10. This means that the penetration distance of medium and large particles would be 53.4 *D* to 69.7 *D* for the real-cough case. For a mouth opening diameter of 2 cm, this means the large particles could penetrate 1 to 1.4 m in the real-cough case, according to our data. This agrees well with the existing studies of large droplet penetration [10, 37]. The deposition of medium and large particles occurs throughout the evolution of the cough jet, as also revealed by Bourouiba et al. [37]. Wei and Li [40] showed that turbulence can significantly enhance the settling range of particles.

### Limitations of this study

One major limitation of this study is that the realistic mouth opening is not circular, and the outlet velocity distribution is intricate due to the complexity of the oral cavity. There is a higher entrainment rate in noncircular jets than round jets due to the three dimensional vortex dynamics [24, 30]. The spread angle of the starting jet airflow in this study was $2\;\tan^{-1}(C_{r,x})\cdot \frac{180}{\pi }=20.8$ degrees, whereas it was 32–38 degrees in the particle image velocimetry measurement on human volunteers in Kwon et al. [13]. By visualizing cough-expired droplet trajectories, Bourouiba et al. [37] showed that droplets at the mouth exhibit a wide range of expiratory directions. The difference in the spread angle may be due to the complex oral cavity, including the possible effects of teeth and head movements during coughing. Furthermore, Bourouiba et al. [37] confirmed the buoyancy force resulting from the temperature difference between the cough flow and ambient flow has a role in changing the trajectory of the cough flow, especially in the interrupted-jet stage. However, the effect of buoyancy force on the flow development and particle transport are not investigated here.

The protocol, which was derived for particles in the Stokes’ region, might have introduced error while mapping the data in water into the realistic situation in air for large particles (Re > 1). Moreover, only the drag and body forces were considered for the particle motion. Wei and Li [40] demonstrated that evaporation has a significant effect on the spread range of medium-sized droplets in a steady cough jet. Although the effect of evaporation is not considered in this study, it is reasonable to infer this effect is not so important in the transient jet, as particles of all sizes have a similar maximum travel distance.

## Conclusions

The two-stage cough jet was experimentally investigated with various boundary conditions. Three different temporal exit velocity profiles—pulsation, sinusoidal and real-cough—were studied. The cough flows in both the starting- and interrupted-jet stages present the self-preserving property. The maximum penetration distances of cough flow are in the 50.6–85.5 *D* range. The real-cough and sinusoidal cases have greater penetrating ability compared with the pulsation cases under the same characteristic Reynolds number and cough expired volume; however, the effects of cough expired volume and Reynolds numbers are more significant, with larger expired volume and Reynolds numbers resulting in further spread of the cough flow.

Scaling relationships were developed to scale particle experiments between the prototype in air and the model in water. The water tank experiments showed that particles of different sizes behaved similarly during the short cough period (0.5 s). They all reached approximately 38 *D* when the cough was stopped (prototype in air: initial velocity 10 m/s, mouth diameter 2 cm, *Q*/*AD* = 250). In the interrupted jet stage, although the medium and large particles were readily deposited, their maximum distance was similar to that of the small particles. The leading vortex played an important role in enhancing the spread range for large particles in particular. The cough duration was important in determining the spread range of particles, and their maximum travel distance was much enhanced in a long starting jet, especially for small particles.
